# Stroke follow-up in primary care: a prospective cohort study on guideline adherence

**DOI:** 10.1186/s12875-018-0872-9

**Published:** 2018-11-28

**Authors:** Rune Aakvik Pedersen, Halfdan Petursson, Irene Hetlevik

**Affiliations:** General Practice Research Unit, Department of Public Health and Nursing, NTNU, Norwegian University of Science and Technology, PO Box 8905 MTFS, N-7491 Trondheim, Norway

**Keywords:** Stroke, Practice guidelines, General practice, Secondary prevention

## Abstract

**Background:**

After a stroke, a person has an increased risk of recurrent strokes. Effective secondary prevention can provide significant gains in the form of reduced disability and mortality. While considerable efforts have been made to provide high quality acute treatment of stroke, there has been less focus on the follow-up in general practice after the stroke. One strategy for the implementation of high quality, evidence-based treatment is the development and distribution of clinical guidelines. However, from similar fields of practice, we know that guidelines are often not adhered to. The purpose of this study was to investigate to what degree patients who have suffered a stroke are followed up in general practice, if recommendations in the national guidelines are followed, and if patients achieve the treatment goals recommended in the guidelines.

**Methods:**

The study included patients with cerebral infarction identified by the ICD-10 discharge diagnoses I63.0 trough I63.9 in two Norwegian local hospitals. In total 51 patients participated. They were listed with general practitioners in 18 different clinics. The material consists of the general practitioners’ (GPs’) medical records for these patients in the first year of follow-up; in total 381 consultations.

**Results:**

Of the 381 consultations during the first year of follow-up, 71 (19%) had stroke as the main topic. The blood pressure (BP) target value < 140/90 mmHg was reached by 24 patients (47%). The low density lipoprotein (LDL) cholesterol target value < 2.0 mmol/L was reached by 14 (27%) of the 51 patients. In total six patients (12%) got advice on physical activity and three (6%) received dietary advice. No advice about alcohol consumption was recorded.

**Conclusions:**

The findings support earlier claims that the development and distribution of guidelines alone is not enough to implement a certain practice. Despite being a serious condition, stroke gets limited attention in the first year of follow-up in general practice. This can be explained by the complexity of general practice, where even a serious condition loses the competition for attention to other apparently equally important issues.

**Electronic supplementary material:**

The online version of this article (10.1186/s12875-018-0872-9) contains supplementary material, which is available to authorized users.

## Background

### Stroke

Ischemic stroke is a frequent disorder with extensive personal and social consequences. In the Western world, stroke is regarded the third most common cause of death [1]. In Norway, about 15,000 persons suffer a stroke each year, and a 50% increase is expected in the period 2010–2030 [2]. The average cost of an acute ischemic stroke hospitalization in Europe has been estimated to $11,900 (2013) and one-year follow-up is estimated to an average of $3720 [3]. Patients with recurrent strokes have more adverse clinical outcomes and costs are higher compared with patients suffering first-ever strokes [4].

### Recurrent stroke and secondary prevention

Having had a first stroke, a person has an increased risk of recurrent strokes; 11% within 1 year, 26% within 5 years and 39% within 10 years. The numbers refer to all strokes [5]. The mortality after a recurrent stroke is particularly high [6, 7].

Risk factors for recurrent cerebrovascular events are well known. Handling these with effective individualized secondary prevention can provide substantial gains in preventing cerebrovascular disease and death [8]. Based on estimates of the benefits from preventive measures for heart attack, it has been suggested that the various secondary preventive measures in combination can reduce the risk of recurrent stroke with up to 70% [1].

Adherence to recommendations for such secondary prevention and to offer the patients the necessary follow-up is a challenge for the health services [8]. Although management of secondary prevention for stroke and transient ischemic attack (TIA) may differ [9], reports on follow-up of TIA may indicate that many patients do not receive secondary prevention at all [10]. Considerable under-treatment with statins in patients with prior cardiovascular disease is documented [11] as is diminishing compliance over time [11, 12]. One answer to such challenges has been development and implementation of clinical guidelines.

### Follow-up in primary care

General practice is well placed to provide follow-up of stroke patients, but this potential is not necessarily fulfilled [13]. Norwegian National guidelines for treatment and rehabilitation of stroke patients [2] were issued in 2010. According to these guidelines, the general practitioner (GP) is supposed to play a key role in the follow-up of patients who have suffered a stroke. Furthermore, the guidelines provide specific advice on the content of the follow-up. It is recommended that patients with stroke should usually be treated with lipid-lowering medication in the form of statins, and that creatine kinase (CK) and transaminase blood samples are taken to control possible side-effects of this treatment. The recommended target value for low-density lipoprotein (LDL) is < 2.0 mmol/L. For blood pressure (BP), the recommended target value is < 140/90 mmHg. Diet, body mass index (BMI), physical activity, alcohol consumption and smoking affect the risk for stroke and recurrent stroke. These lifestyle factors should therefore also be part of the post-stroke follow-up. In the Netherlands, it was found that advice in a protocol corresponding to the Norwegian guidelines is followed only to some extent. As an example, lifestyle advice was offered to only one in four patients [14]. After the introduction of the Dutch protocol, only minimal impact was noted on clinical practice and no major changes in survival or secondary outcomes were found [15].

However, it is still not known to what degree the patients are followed up in general practice in Norway, nor is it known if the follow-up is in accordance with the guidelines. It is known that development and distribution of guidelines alone is not sufficient for implementation in general practice [16]. It is therefore recommended to make use of more active methods for implementation [16], but even comprehensive active implementation does not necessarily lead to adherence to the clinical guidelines [17, 18].

In this study, the aim was to investigate the extent to which patients who have had a stroke are being followed up in general practice, if the recommended procedures have been applied and whether or not patients achieve the treatment goals recommended in the national guidelines.

## Methods

### Design and setting

The study took place in Møre og Romsdal County in Western Norway. This is an affluent area with good access to primary and secondary health care. In 2016 the county had the highest life expectancy for boys and the second highest for girls among all 19 Norwegian counties [19]. In Norway, all residents are entitled to a regular general practitioner (RGP). When a resident is registered on a GP’s list of patients, the GP has the medical responsibility for this person. At the time of this study, about 99% of the Norwegian population was registered on GPs’ patient lists [20]. When a person registered on a GP’s list is discharged from hospital, the GP normally receives a discharge summary. The discharge summary is a transfer of information from the hospital to the GP, not a formal referral. The guidelines [2] state that discharge summaries of good quality sent to the RGP is a prerequisite for the follow-up of stroke patients.

The study included patients treated for ischemic stroke in two Norwegian local hospitals in 2011 and 2012. Patients with hemorrhagic stroke were not included. The reason for this was that the guidelines do not apply to all forms of hemorrhagic stroke. A search for the ICD-10 discharge diagnosis I63.0 trough I63.9 identified patients with cerebral infarction in the hospital files. The patients’ RGPs were identified by The Norwegian Health Economics Administration (Helfo). All patients identified in the hospital files were registered with an RGP, and all patients included had active practicing RGPs in clinics with regular office hours and availability. All clinics were available for wheelchair users, and all clinics had secretaries available by phone. All clinics also had laboratory services including availability of blood investigations such as CK, transaminases and cholesterol. The costs of laboratory services are covered by the National Insurance Scheme. Residents in Norway have compulsory membership in this scheme. None of the clinics had dietitians or rehabilitation therapists as part of their staff.

Invitation to participate in the study was sent to each of the GPs identified as described above. Only patients living in their own home and registered with an RGP who accepted participation, were subsequently invited to participate in the study. Patients in nursing homes were excluded.

One of the authors (RAaP) visited each clinic personally. All clinics kept electronic medical records, and each clinic provided access for the researcher. The GPs used three different electronic medical record systems. We evaluated the records of each consultation (*n* = 381) in the RGPs’ clinic the first year after the hospital stay or the last outpatient hospital consultation. The record’s laboratory results, prescribing registries and diagnosis registries were all used to support the evaluation of the written text record of each individual consultation. An operational definitions list was used to standardize the coding of data (Additional file 1).

We noted the number of consultations with any content relevant for stroke follow-up, as was the number of consultations mainly concerning stroke. A note was made where we found that any of the lifestyle factors diet, BMI, physical activity, alcohol consumption or smoking had been addressed in the consultation. We chose to include those who had a recorded BMI, also when recorded before the specific follow-up year. Furthermore, we recorded whether or not the recommended blood tests were taken and the results of blood pressure measurements and LDL laboratory results, as these tests have specified targets in the guideline, expected to be reached.

## Results

Among 100 invited GPs, 37 agreed to participate. These 37 GPs had a total of 138 stroke patients from 2011 and 2012 on their lists. We invited all these 138 patients to participate in the study, and 51 gave their written consent. Age varied from 38 to 90 years (mean 68.5 years). Thirty (59%) were male and 21 (41%) female. These 51 patients had RGPs in 18 different clinics.

In total 46 patients (90%) had suffered from an acute stroke. Among these, 40 had their first-ever stroke, and 6 had a recurrent stroke. The rest of the patients in the total group of 51 patients had TIA following a previous stroke or previous stroke with new symptoms not classified as TIA, and where new stroke could not be detected. In one patient, we found that the stroke diagnosis was used, but not further discussed in the discharge summary. Five patients got acute thrombolytic treatment and 19 of the patients were discharged with an outpatient control appointment.

### Consultations

These 51 patients had 381 consultations with their RGP the first year after discharge from hospital, an average of 7.5 (0–24) consultations.

### Stroke follow-up

In 148 consultations (39%), stroke was documented as a topic. We found that 71 (19%) of the consultations had stroke as the main topic. The medical record in these cases was primarily concerned with stroke, although other issues also were discussed. On average, each patient had 1.4 (0–7) consultations mainly concerning stroke during the first year of follow up in general practice.

### Adherence to the most central advice for the follow-up

Table 1 gives information about the number (percentage) of relevant lifestyle information registered in the patient records, as well as procedures performed (BP and LDL-cholesterol) in addition to the number of patients where the recommended goals were reached.

**Table 1:** Variables registered and goals reached

	Women (*n* = 21)		Men (*n* = 30)		Total (*n* = 51)	
	N	%	n	%	n	%
*Variables registered*						
Lifestyle^a^	7	33	9	30	16	31
BMI^b^	6	29	6	20	12	24
Diet^c^	2	10	1	3	3	6
Physical activity^d^	4	19	2	7	6	12
Smoking^e^	1	5	3	10	4	8
Alcohol^f^	0	0	0	0	0	0
BP^j^	20	95	26	87	46	90
LDL^h^	13	62	15	50	28	55
*Goals reached*						
BP^i^	11	52	13	43	24	47
LDL^j^	7	33	7	23	14	27

^a^Patients with any notes on lifestyle made the first year of follow-up. ^b^Patients with a BMI measure made before or the first year after the stroke. ^c^Any notes on diet during the first year of follow-up. ^d^Any notes on physical activity the first year of follow-up. ^e^Any notes on smoking the first year of follow-up. ^f^Any notes on alcohol consumption the first year of follow-up. ^j^Blood pressure measured the first year of follow-up. ^h^LDL measured the first year of follow up. ^i^BP < 140/90 mmHg in the last registration in the study period. ^j^LDL < 2.0 mmol/L

### Other recommendations

On discharge from hospital, 45 of the 51 patients were treated with statins. In the GPs’ medical records, we found confirmation of ongoing statin treatment in 39 patients. In our material, CK and transaminase blood samples were taken in 8 (16%) of the 51 patients, and in 7 of the 39 patients where we could find confirming information about ongoing statin treatment in the GPs’ medical records.

## Discussion

### Main findings

In this study we examined the follow-up of patients with stroke in general practice. We compared the recommendations in the national guidelines with clinical practice in real life. Nearly all patients had their blood pressure measured within the first year of follow-up in general practice. Despite this, only about half of the patients had reached the blood pressure target value. Fewer patients had their LDL-cholesterol level measured, and the LDL-cholesterol target value was reached by less than one third of the patients. We found limited information on diet and physical activity and none about alcohol consumption in the medical records.

Patients residing in the community had on average consulted their GP more than seven times the first year after being discharged from hospital with a stroke diagnosis. In 2012, Norwegians on average consulted their RGP 2.6 times [21]. However, in our material we also found that stroke was not necessarily the foremost concern in the consultations with the GP in this period. In fact, stroke was not documented as an issue at all in most of these consultations. The findings reveal that the median number of consultations mainly concerning stroke was one in the first year of follow up. Stroke got limited attention in the consultations with the GP the first year after the incident, even though it is a serious condition. This may be because the stroke diagnosis faced competition from other issues that were perceived as equally important by the patient or by the GP at the moment of consultation.

The guidelines give evidence-based advice on the follow-up of stroke survivors. They are based on a thorough review of an extensive amount of research and are intended to ensure good clinical practice. They can be regarded as a map that gives direction and guides the most important clinical decisions for the treatment of patients who have experienced stroke. Our study, however, shows that this map is not in accordance with the terrain in general practice.

### Strengths and weaknesses

Based on the GPs’ medical records, this study investigates how the follow-ups of the patients have been recorded. It does not involve any form of possible biased self-reporting. The inclusion of patients started out wide, and although the participation rate was low, the researchers did not make any selections. All data collection was done by the same person. In this way, there were no different practices in the review of the medical records or data registration. The possibility of intra-observer variations is nevertheless still present.

We found a low degree of adherence to the guideline. We have no reason to believe that the GPs who accepted participation in this study have a less optimal practice than those who rejected the request. On the other hand, we have some indication of the opposite. When we made reminding telephone calls to all the invited GPs that did not respond to our invitation letters one GP admitted that it was scary to have her practice investigated by researchers.

Still, there are several limitations to this study. There were relatively few patients included, and although the GPs are practicing in 18 different clinics, the clinics are all in the same county. In addition to a low rate of participation among of GPs, there was also a low degree of participation among patients. Possible explanations for this could be poor health, high age and impaired physical and mental functioning in the patients. We needed, for example, to exclude a patient because this persons’ partner had signed a declaration of consent, without documentable transfer of authority for consent. The experienced treatment burden among those with stroke is shown to be considerable [22] and combined with the reduced patient capacity, this may also be the reason for the low degree of participation among the invited patients. While the chosen method of reading medical records has its strengths, it also carries with it some weaknesses. There is a possibility of making incorrect recordings. To check this, one would have to visit the clinics again to read the records once more. Given the geographical spread, this would be very resource-demanding.

Furthermore, physicians do not necessarily document every topic of the consultation. Therefore, it is possible that the GP or the patient have addressed topics without it being included in our counts. Despite these weaknesses, we claim the findings to be valid for the performance of secondary stroke-prevention in general practice.

### Findings in the light of current knowledge

We have found that the patients often consult their GPs in the period after having had a stroke. In this way, our study is consistent with previous claims that general practice is well placed for the follow-up of patients who have had a stroke [12]. The reasons for lack of guideline adherence are largely unknown. It could be that the GPs do not know the recommendations for secondary prevention after stroke [16], that the guidelines do not fit in with the patients` complexity [23] or that the guidelines are poorly adapted to general practice in other ways [24]. There are also critical questions as to whether the theoretical basis for clinical guidelines is good enough as guidelines are mostly organ-specific. This results in a high degree of complexity in general practice where patients often have many diseases at the same time [25]. International expert guidelines are also documented to be non-implementable in Norwegian general practice because of the resource utilization recommended is not compatible with the resources available. For example, international guidelines on high blood pressure alone have been estimated to impose a workload that exceeds the total working capacity of Norwegian GPs [26]. To the extent that the guidelines are followed with respect to recommended procedures being carried out, it is nevertheless a recognized problem that this does not have to significantly affect health goals for patients [17, 18].

It is known that the risk of stroke can be reduced by adjustments in lifestyle and that lack of knowledge is a main obstacle for patients in achieving this benefit [27]. The guidelines [2] are explicit on lifestyle advice; they give recommendations on physical activity, diet and alcohol consumption in addition to BMI. One prominent finding in this study is the absence of such lifestyle advice, and especially advice on diet and alcohol consumption. This corresponds to previous findings, pointing out that patients report having received little or no information about lifestyle following a stroke [28].

We recommend future research to explore reasons for non- adherence to guidelines. Other studies point out that the majority of patients with a chronic disease in primary care, also have other chronic diseases; multimorbidity [29]. Also, most persons aged 65 and older are multimorbid [30, 31]. In our research group, we will therefore conduct further research on stroke follow-up in the presence of multimorbidity. General practice is not meant to focus on one single disease, but practices an integrated approach. An understanding of this complexity might give answers to why the follow-up of patients who have had a stroke seems to be so limited in general practice.

## Conclusions

Although patients frequently consulted their GPs in the first year after a stroke, most consultations were concerned with issues other than the stroke. When stroke was an issue, the recommendations in the guidelines were often not adhered to. This means that even a medical condition considered to be serious, may receive only limited attention in general practice. As the access to the GP did not seem to be limited, the results may rather be caused by the complexity of general practice. Other complaints may be regarded as equally important by the patient or by the GP. This complexity should be considered in the development of clinical guidelines.

## Additional file

Additional file 1: Operational definitions list. List of definitions used when registering data from the medical records. (DOCX 13 kb)

## Abbreviations

BMI: Body mass index
BP: Blood pressure
CK: Creatine kinase
GP: General practitioner
Helfo: The Norwegian Health Economics Administration
LDL: Low-density lipoprotein
REK: Regional committees for medical and health research ethics
RGP: Regular general practitioner
TIA: Transient ischemic attack
