# Supplementary material for: Stroke follow-up in primary care: a prospective cohort study on guideline adherence
Source: BMC Fam Pract. 2018 Nov 28;19:179. doi: 10.1186/s12875-018-0872-9 (PMC6263549; doi:10.1186/s12875-018-0872-9)
Supplement: Supplementary file 1 — Operational definitions list. List of definitions used when registering data from the medical records. (DOCX 13 kb) [file 12875_2018_872_MOESM1_ESM.docx]

**Operational definitions list**

1. **Study period:**

Read date of last outpatient control for stroke. Read date of discharge if no outpatient control was made.

1. **Number of consultations:**

All consultations: Read number of all consultations the first year after the last outpatient control or the first year after discharge if no outpatient appointment was made.

Consultations with stroke as a topic: Count any consultation where stroke is mentioned in the medical records. Do not count a consultation where a measurement relevant to stroke is made without it assessed in the context of stroke. E.g: BP measurement outside the context of stroke is not counted as a consultation with stroke as a topic.

Consultations with stroke as the main topic: Assess the written text of the medical record for the consultation. Count consultations where stroke is the most prominent issue, do also count consultations where stroke is one of two prominent issues and it is reason to believe that at least half of the time spent in the consultation was occupied with the issue of stroke.

1. **Lifestyle advice**

Period of study: from discharge to one year after last outpatient control for stroke.

Diet: Read any advice on diet recorded.

Physical activity: Read any advice on physical activity recorded.

Smoking: Read any advice on smoking recorded.

Alcohol: Read any note on alcohol recorded

BMI: Read any registration of BMI recorded. Extended period: Any time up to one year after last outpatient control. Include before the stroke.

1. **Recommended blood tests**

Period: One year from last outpatient control or one year from discharge if no outpatient appointment for stroke was made.

LDL: Read any LDL measurement in the period.

ASAT: Read any ASAT measurement in the period.

ALAT: Read any ALAT measurement in the period.

CK: Read any CK measurement in the period.

1. **Blood pressure**

Period: One year from last outpatient control or one year from discharge if no outpatient appointment for stroke was made.

BP: Make note of all blood pressure measurements in the period.

1. **Goals reached**

BP: Assess the last recording in the period. If this measurement shows BT < 140/90 mmHg, goal is reached.

LDL: Assess the last recording in the period. If this measurement shows LDL<2,0 mmol/L, goal is reached.
